# Making Sense of the Complexity of Decentralised Governance: Comment on "The Effects of Health Sector Fiscal Decentralisation on Availability, Accessibility, and Utilisation of Healthcare Services: A Panel Data Analysis"

**DOI:** 10.34172/ijhpm.2022.7449

**Published:** 2023-01-01

**Authors:** Seye Abimbola

**Affiliations:** School of Public Health, Faculty of Medicine and Health, The University of Sydney, Sydney, NSW, Australia.

**Keywords:** Governance, Realist, Decentralisation, Complex Intervention, Evaluation, Health Systems

## Abstract

The article by Rotulo and colleagues suggests that health sector fiscal decentralisation has been bad for Italy. But given the complexity of fiscal decentralisation, this interpretation is not necessarily so. Their analysis was based on assumptions about causality that are better suited for simple interventions. Assumptions of simplicity show up as misleading artefacts in the conclusion of evaluations of complex interventions. Complex interventions work by triggering mechanisms – eg, reasoning and learning processes – that manifest differently across the units of a decentralised system, contingent on context, evolving over time. Evaluation findings can only be partial and provisional; neither summarily good nor bad. The goal of evaluating a complex intervention – such as decentralised governance – should be to understand how, under what circumstances and for whom they are good or bad – at a point in time.

 What does one make of the findings of the evaluation of a complex intervention?^1^ You may assume that the findings say something complete and final. Or you may assume that whatever the findings say about the impacts of a complex intervention are inevitably partial and provisional. Each of these two epistemic assumptions leads to different approaches to evaluation.^2^ In their evaluation of the effects of health sector fiscal decentralisation in Italy, Rotulo and colleagues^3^ seem to have started with the former assumption – that it is possible to say something complete and final about whether a complex intervention such as decentralised governance is good or bad. One’s starting epistemic assumption about the evaluation of a complex intervention has implications for one’s research questions or interpretation of findings.^2^ Whatever one’s starting epistemic assumptions, interpreting the findings of the evaluation of any complex intervention requires careful judgement.

 A distinguishing feature of complex interventions is that they rely on human reasoning to enact their effects.^2^ Their effects are always evolving, contingent on learning^4^ – ie, on making the link ‘between past actions, the effectiveness of those actions, and future actions.’^5^ The findings of their evaluation are inevitably provisional – what was true last year may not be true this year. Another distinguishing feature of complex interventions is that they work differently for different people or units within a system. Each may have a different experience. The truth is not to be found in simple aggregates.^6^ In the case of decentralised governance, the units of the system – such as the regions of Italy – will inevitably have differing experiences, individually or in relation to one another.^2^ A national aggregate cannot do justice to the experience of each unit. It is inevitably partial – just as the experience of one unit is no substitute for the experience of another unit. One cannot, on the basis of an evaluation, draw any firm conclusion as to whether fiscal decentralisation is good or bad for a country.

 The findings by Rotulo and colleagues that fiscal decentralisation in Italy decreased ‘the availability of staff and hospital beds,’ decreased ‘hospitalisation rates,’ and increased ‘inter-regional patient mobility for health care,’^3^ are therefore unremarkable. The findings say something potentially useful about *how* fiscal decentralisation may have – *so far* – impacted health system equity and efficiency and perhaps resilience (to shocks such as the coronavirus disease 2019 [COVID-19] epidemic) in Italy. But they do not say anything about whether or why fiscal decentralisation is good or bad for Italy. They say much too little about the mechanisms underlying these effects, and what national or sub-national policy response to these findings might be – beyond a wholescale winding back of fiscal decentralisation, or a return to centralised governance of Italy’s national health service. To conclude from these findings that fiscal decentralisation fails to deliver or improve health system equity, efficiency and resilience in Italy or anywhere else is to risk throwing out the baby with the bathwater.

 But first, it is worth knowing if there is a baby in Italy’s fiscal decentralisation bathwater. In other words, a good place to start the inquiry about how fiscal decentralisation has fared – *so far* – in Italy is by examining the mechanisms (ie, the reasoning and learning processes, and the patterns of behaviour of individuals, sub-national units, and the national government) that decentralisation had set in motion.^2^ As Rotulo and colleagues’^3^ effort demonstrates, this is difficult to do in the aggregate – ie, without closely examining the dynamics of sub-national responses, alongside the evolving national response. In a realist synthesis of global literature on how decentralisation affects health system performance (specifically: equity, efficiency and resilience), Abimbola and colleagues^2^ identified three such mechanisms that may be unleashed by decentralised governance:

 “*‘voting with feet’ (reflecting how decentralization exacerbates or assuages the existing patterns of inequities in the distribution [or movement] of people, resources and outcomes in a jurisdiction); ‘close to ground’ (reflecting how bringing governance close to the people allows for use of local initiative, information, feedback, input and control); and ‘watching the watchers’ (reflecting the many mutual accountability relations between multiple centres of governance within a jurisdiction which are multiplied by decentralization, involving governments at different levels and also community-level entities).*”^2^

 Each of these three mechanisms – *‘voting with feet,’ ‘close to ground,’ *and *‘watching the watchers’* –can determine what happens when fiscal decentralisation kicks in within a country such as Italy. Each of the three mechanisms is modulated by a range of contextual factors that influence whether decentralised governance is good or bad for health system equity, efficiency or resilience. Effects of the three mechanisms and the contextual factors that influence them may manifest differently within each sub-national entity or across the entire country.^2^ Using the three mechanisms as lenses to make sense of how a complex intervention such as fiscal decentralisation affects health system performance means one can see the baby and the bathwater with greater clarity.

 Take for example the impact of decentralised governance on equity. Rotulo and colleagues found that fiscal decentralisation ‘has a negative impact on human resources, with the density of all type of staff decreasing,’ which is worse in less wealthy regions; that fiscal decentralisation led to an ‘overall decrease in hospital beds,’ a decrease that is inequitably distributed; and that fiscal decentralisation is associated with an increase in the density of private sector doctors and public sector doctors, in regions with higher incidence of relative poverty.^3^ These findings suggest that fiscal decentralisation has – *so far* – had a negative impact on health system equity in Italy. This may be true, but not necessarily so. The ‘*close to ground*’ mechanism suggests that with decentralisation, there is greater use of local information in resource allocation, such that sub-national governments may choose to invest more in equity-promoting health services and relatively less in curative services.^2^ Such pattern of resource reallocation may have occurred, at least in part in Italy, given that Rotulo and colleagues also found that ‘regional surpluses play a negative effect on hospital beds density… suggesting that regions with more fiscal space do not necessarily invest more in hospital resources.’^3^

 As an example of how decentralised governance affected efficiency, consider Rotulo and colleagues’ finding that fiscal decentralisation decreased ‘the share of residents to total patients’ within regions, therefore ‘suggesting an increase in the share of patients moving from one region to another to seek treatment.’^3^ They cited a previous study in Italy^7^ that shows such mobility, ‘especially from regions with historically weaker healthcare services’^3^ – a pattern that is in keeping with the ‘*voting with feet*’ mechanism. But what also needs to be acknowledged are previous analyses in Italy^7^ and elsewhere^8^ suggesting that poor sub-national units with wealthy neighbouring units can reap efficiency gains by strategically under-investing in selected health services in anticipation of their residents’ use of cross-border services, or by formally outsourcing selected services to such neighbouring units.^2^ Even then, this pattern of behaviour in response to fiscal decentralisation will vary depending on whether poor units have wealthy neighbours, the capacity of such units to make strategic decisions, or the willingness of wealthy neighbours to enter into ‘outsourcing’ arrangements.^7,9-11^ These contextual possibilities will evolve over time, and are contingent on learning. A snapshot is no final word.

 In drawing the policy implications of their findings, Rotulo and colleagues argue that ‘Italy’s capacity to effectively manage and control the COVID-19 epidemic in 2020 suffered from low availability of resources, fragmentation of services, weak public provision, and spatial disparities’ – all, according to Rotulo and colleagues, ‘factors that fiscal decentralisation has exacerbated.’^3^ This argument raises two issues related to how one makes sense of the complexity of decentralised governance – both of them linked to the ‘*watching the watchers*’ mechanism. First, in Italy as elsewhere, the national government retains residual roles which include aspects of epidemic preparedness and response.^12^ As such, rather than fiscal decentralisation, Italy’s COVID-19 response may have more to do with the extent to which the national government used its levers to influence sub-national units^12^ or with long-running austerity measures.^13^ Second, it is not inevitable that fragmentation will lead to worse outcomes. Fragmentation can also limit the spread of epidemics, increase the likelihood of learning from other unit’s response, while also making local course-correction easier^14^ – all of which played out, for example, in the COVID-19 response in Australia, another fiscally decentralised country.^15^

 As for every complex intervention, it is hard to disentangle context from mechanism and ultimately from impact. It is, as a result, difficult to go from the findings of Rotulo and colleagues’ evaluation to policy advice. It is one thing for specific mechanisms to be influenced by contextual factors in the same or different ways. It is another thing for all the three mechanisms to operate against a broader background of long-running change, as is the case in Italy. Somewhat midway through Rotulo and colleagues’ study period (2001-2017), Italy’s long-running austerity measures were worsened by the Great Recession (2008),^13^ and a major national political upheaval (2011).^16^ Combine these with long-running trends in health policy and practice in Italy and other high-income countries towards de-hospitalisation, managerialism and growing public expectation of ready access to high-end hospital equipment.^17^ It is not possible to make sense of the impact of fiscal decentralisation on the health system in Italy without taking these long-running trends and changes seriously.

 An evaluation that begins by examining the dynamics of how the three mechanisms are playing out in Italy or elsewhere would provide richer accounts of the impact of decentralised governance on the health system. Such evaluations would require additional data, including qualitative data – eg, to study the learning processes triggered by decentralised governance.^18^ Rotulo and colleagues^3^ may have worked within the constraints of available data to answer questions the data allowed them to ask. Even then, the data available to Rotulo and colleagues^3^ may have still been sufficient to answer some mechanism-driven questions – given the authors’ passing references to these mechanisms as they sought to make sense of their findings. The focus of future research in Italy and elsewhere should now be to find, collect, and curate the kind of (qualitative and quantitative) data that would allow scholars to ask mechanism-driven questions and to test or refine theory-based assumptions – as the starting point of inquiry on the impacts of decentralised governance on health systems.

 While interpreting the findings of the evaluation of any complex intervention, it is important to seek out alternative explanations, which may suggest – in the case of fiscal decentralisation in Italy – that there is a baby in the bathwater. One must consider what this baby might be in any evaluation of the impacts of decentralised governance on health system performance. The impacts of decentralised governance are always mixed – there is both good and bad.^2^ The least one can ask of any evaluation is to provide insights on what national or sub-national decision-makers must do to assuage the negative impacts of decentralised governance or to accentuate its positive impacts^2^ – whatever they might be. We must ask: What is good, what is bad, and under what circumstances are they good or bad? Without such a clear sense of ‘circumstances’ tied to mechanisms, one is at a loss as to what decisions to make in response to evaluation findings, especially of complex interventions.

## Ethical issues

 Not applicable.

## Competing interests

 Author declares that he has no competing interests.

## Author’s contribution

 SA is the single author of the paper.
